# Multi Response Optimization of ECDM Process for Generating Micro Holes in CFRP Composite Using TOPSIS Methodology

**DOI:** 10.3390/polym14235291

**Published:** 2022-12-03

**Authors:** Manpreet Singh, Sarbjit Singh, Jatinder Kaur Arora, Parvesh Antil, Ankit D. Oza, Dumitru Doru Burduhos-Nergis, Diana Petronela Burduhos-Nergis

**Affiliations:** 1 Punjab State Council for Science and Technology, Chandigarh 160019, India; 2 Department of Mechanical Engineering, Punjab Engineering College, Chandigarh 160012, India; 3 Department of Basic Engineering, College of Agricultural Engineering & Technology, CCS HAU, Hisar 125004, Haryana, India; 4 Department of Computer Sciences and Engineering, Institute of Advanced Research, Gandhinagar 382426, Gujarat, India; 5 Faculty of Materials Science and Engineering, Gheorghe Asachi Technical University of Iasi, 700050 Iasi, Romania

**Keywords:** carbon fiber reinforced polymer composite, electrochemical discharge machining, Taguchi’s method, TOPSIS, micro-holes, morphology

## Abstract

The applications of carbon fiber reinforced polymer composites (CFRPCs) in aerospace, automotive, electronics and lab-on-chip devices require precise machining processes. Over the past decade, there have been numerous attempts to machine CFRPCs using both traditional and unconventional machining techniques. However, because of their limitations, these methods have not gained widespread acceptance. In the present research investigation, Electrochemical Discharge Machining (ECDM) process has been employed to produce micro-holes on CFRPC. The experimental strategy was scheduled using L_9_ orthogonal array keeping applied voltage, electrolyte concentration and inter-electrode gap as input parameters. The material removal rate (MRR) and overcut were selected as output parameters. The technique for order preference by similarity to the ideal solution (TOPSIS) methodology was executed for multi-response optimization. The overcut and MRR of machined samples improved from 150 µm to 48 µm and 2.232 mg/min to 2.1267 mg/min correspondingly while using the optimum parametric settings of the TOPSIS approach. The shape of drilled micro-holes produced by the TOPSIS process is indicative of a machined surface of superior quality, with a reduction in the number of micro-cracks and a diameter that is uniform.

## 1. Introduction

**Figure 1:**
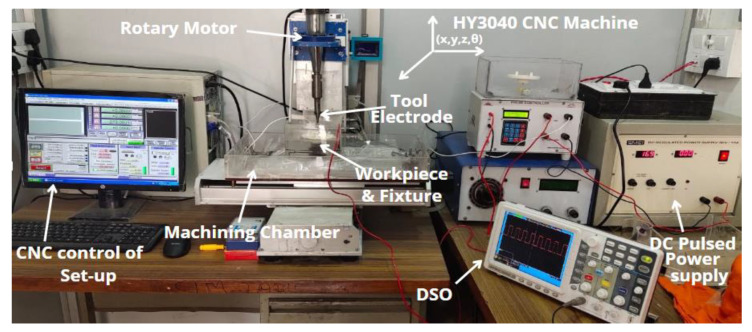
Pictorial view of ECDM setup.

Carbon fiber-reinforced polymer composites (CFRPCs) are gaining importance in various industrial sectors like aviation, electronics, electromagnetic interference shielding and lab-on-chip devices because of their superior mechanical properties [1,2,3]. Modern industry demands the fabrication of micro-holes on special or advanced materials for microreactors and microfluidic applications. The existing machining processes whether conventional or non-conventional are practically not capable to machine CFRP composite due to their fibrous and semi-conductive nature. In the domain of conventional machining processes, the drilling and milling process were widely used for processing of CFRP though it remains less popular owing to delamination, tool wear and uncut fibers [4,5]. On the other hand, the Electrical Discharge Machining (EDM) has gained the interest of research fraternity for surface modification and micro-machining of CFRP [6,7]. Though, the application of EDM process was limited for CFRP composites due to its partial conductivity. Later on, Electrochemical Discharge Machining (ECDM) process suitably addresses all these machining constraints with respect to CFRP or other fibrous materials [8,9]. The pictorial view of the ECDM setup is shown in Figure 1. The process of spark generation and machining is as follows: the regulation of electrode potential initiates the electrolysis process and generates a hydrogen layer on the surface of the tool electrode [10]. This hydrogen layer act as an insulator between cathode and the electrolyte. As the electrode potential increases beyond a critical value, the breakdown of gas film causes spark generation at the tip and periphery of the cathode. A gap of few microns is essential between cathode and workpiece for the generation of a uniform spark at the tip of the cathode. The induced discharge causes the removal of material from the workpiece surface due to melting, vaporization, and chemical etching [11,12].

In recent years, researchers attempted drilling and slicing of conductive and non-conductive materials using the ECDM process. Wuthrich et al. [13] drilled micro-holes on glass and concluded that machined surface quality becomes poor at higher machining depths. Sarkar et al. [14] attempted micro-machining of silicon nitride ceramic and observed that material removal is dominantly affected by the applied voltage. Similar behavior has been observed by Huang et al. [15] during generating micro holes on a stainless steel plate. Liu et al. [16] incorporated grinding action in the ECDM process for the micromachining of metal matrix composite (MMC). Later on, Jha et al. [17] successfully cut micro-slots on MMC using an abrasive particle-coated disc irrespective of the tool electrode in the ECDM process. Antil et al. [18,19] analyzed the behavior of ECDM process parameters in the machining of the fibrous polymer composite and concluded that the quality of machined hole walls becomes poor, owing to cracks and uneven heating. During the machining of carbon fiber epoxy composite, Singh et al. [20] concluded the hat quality of drilled blind holes was poor due to uncontrolled discharge rate with a variety of process parameters. Yan et al. [21] studied the machining of super-alloys with the incorporation of the tubular electrode in the ECDM process. From the extensive literature survey, it has been observed that there is limited information available micromachining of fibrous materials, especially CFRP materials using the ECDM process.

The superiority of machining processes depends on the optimum selection of process parameters. For assessing the excellence of machining processes, there are various techniques and methods for optimization of multi-response problems such as Grey Relational Analysis (GRA), Analytic Hierarchy Process (AHP), Response Surface Methodology (RSM), Genetic Algorithm (GA) and Technique for Order Preference by Similarity to Ideal Solution (TOPSIS) etc. Among these techniques, the TOPSIS method has gained popularity due to its simple computation and application in different fields such as mathematics, manufacturing, economics and information systems etc. [22]. The TOPSIS method shows that the optimum solution is farthest from the worst and nearest to the positive solution. Nayak and Mahapatra [23] successfully optimized quality characteristics in the wire EDM process by grouping of AHP and TOPSIS methods. Nguyen et al. [24] implemented combined Taguchi-TOPSIS techniques for multi-response optimization of EDM process. Parthiban et al. [25] optimized process parameters for Laser machining of alloys by TOPSIS method. Ladeesh and Manu [26] used the TOPSIS method for optimization of quality characteristics in grinding assisted ECDM of glass. Kumar et al. [27] successfully used RSM & TOPSIS method for the best selection of output quality characteristics in plasma arc machining process.

**Figure 2:**
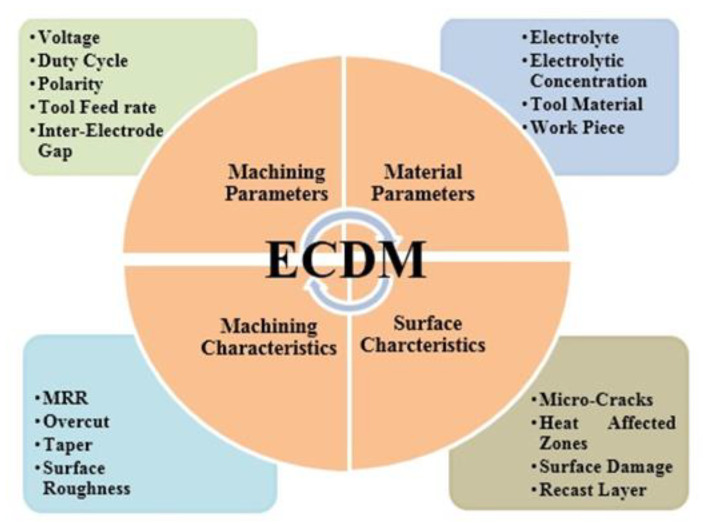
Process variables and output quality characteristics.

The behaviour of any machining process depends upon its input process parameters and output performance characteristics. The selection of input parameters and their level is a cumbersome job and requires a scientific and judicious approach. The input process parameters of ECDM are categorized into two categories i.e., (1) Machining Parameters, (2) Material Parameters; as represented in Figure 2. The machining parameters i.e., applied voltage, duty cycle and polarity were related to supply of discharge energy to machining regime and inter-electrode gap deals with distance between tool electrode and auxiliary electrode of ECDM process. Moreover, the material parameters i.e., nature of electrolyte (alkaline, acidic or neutral), electrolyte concentration (affects the surface characteristics), tool electrode material (mechanical, thermal and erosion resistant properties) and workpiece (conductive or Nonconductive) plays a significant role on the output quality characteristics of the machined materials. Conversely, the output quality characteristics of ECDM are categorized into two categories i.e., (1) Machining characteristics; (2) Surface characteristics. The machining characteristics include material removal rate (MRR), surface roughness, overcut and taper. When the researches are talking about micro machining, the surface finish, overcut and taper are more prominent than MRR. The surface characteristics include micro-cracks, Heat Affected Zones (HAZs), surface damage on machined surface, recast layer formation etc. There exists an intricate connection between input and output quality characteristics of ECDM process, as shown in Figure 2. The machining of semi-conductive and fibrous materials has various challenges in dealings with output quality characteristics. Therefore, multi-response optimization is a necessary step for better output quality characteristics of ECDM process.

## 2. Motivation and Problem Formulation

The quality is the primary criteria behind the selection of machined samples and micro-products in various applications such as Micro Electromechanical Systems (MEMS), lab on chip devices and in defence sector etc. During machining of polymer based composites, the output quality characteristics are not up to mark. Therefore, the research fraternity is working on improvement of the quality characteristics of polymer based composites. Moreover, high productivity with better surface characteristics enforces various limitations in machining due to their intricate relationship. Therefore, multi-response optimization is important for achieving the desired output quality characteristics. More importantly, the machining of CFRP composite using ECDM process is rarely informed in the research literature. With these practicalities, the problem is formulated in this research work. The numeric values of input process parameters were selected on the basis of one factor at a time (OFAT) approach. The machining process parameters of ECDM are presented in Table 1.

## 3. Experimentation Strategy

The CFRP composite was used as workpiece material for the experimental analysis. The weight distribution of weft and warp carbon was equally shared with positioning of 0/90^0^. The CFRP laminate of 2 mm thickness was used for experimentation. The micro-machining of CFRP composite was performed on advanced ECDM set up integrated with five axis HY3040 CNC machine as shown in Figure 1. The NaOH electrolyte was selected among various electrolytes because of better machining performance [9]. The tungsten carbide drill bit having diameter 200 microns was used for experimentation. The obtained results for output characteristics using L9 orthogonal array as per Taguchi’s methodology are shown in Table 2. The MRR of machined samples were measured using micro weighing balance of accuracy ±0.001 mg. The quality & overcut of hole was initially analysed using Carl Zeiss microscope (Model-Stemi-508) and further validated by Scanning Electron Microscopy (SEM, Model-JEOL JSM-6510LV). For, measuring overcut of hole, the Image J software was used.

## 4. Results and Discussion

### 4.1. Material Removal Rate (MRR)

**Figure 3:**
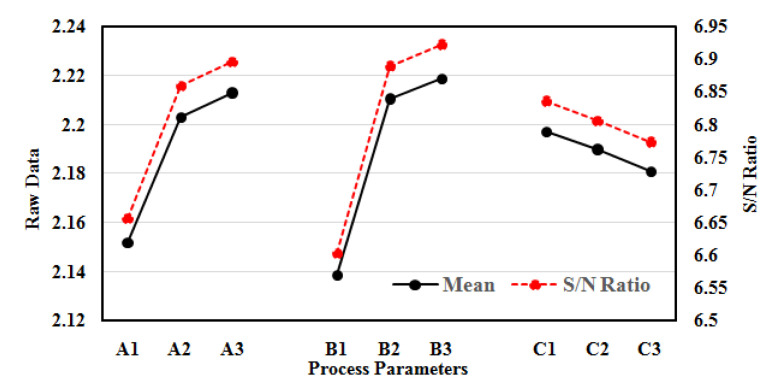
Raw data and S/N ratio plot for MRR.

The behaviour of MRR with a variation of process parameters is presented in Figure 3. The MRR increases with the increase in voltage from level A1 to A2 (45 V to 55 V) and from A2 to A3 (55 to 65 V), but from stage A2 to A3 a slight decrease in MRR is observed as compared to A1 to A2. Therefore, it appears from the figure that MRR escalates with an escalation in voltage. Although, this increase is not uniform in nature, but the prime reason for this increase is an increase in spark energy in machining regime. The increase in voltage accelerates the formation of hydrogen bubbles during the electrolysis process and leads to the development of thicker hydrogen bubble casing at the tool electrode surface. Therefore, the thick hydrogen layer at the tool electrode surface produces spark energy of high intensity. On the other hand, at a higher voltage from level A2 to A3 (55 to 65 V), there is an increase in spark intensity but the MRR becomes lower due to entrapment of debris in the machining regime. The similar finding has been observed in machining ZrO_2_ ceramics, and SiC reinforced composite using EDM and ECDM processes, respectively [18,19,28].

Similarly, the MRR significantly escalates with the escalation of electrolyte concentration. The MRR dominantly increases with increase in electrolyte concentration from level B1 to B2 (10% to 20%) and level B2 to B3 (20% to 30%). In comparative, the increase in MRR from level B2 to B3 is slightly lower than level B1 to B2. The increase in concentration of electrolyte strengthens the mobility of electrochemical reactions in the machining zone. The accelerated electrochemical reaction increases the electrical conductivity of the solution and increases the generation of hydrogen bubbles in the vicinity of tool electrode. These dense hydrogen bubbles combine to form thick hydrogen layer on the periphery of tool electrode. This tends to generate spark energy of high intensity on the surface of tool electrode. At higher electrolyte concentration from level B2 to B3 (20% to 30%), the MRR slightly increases due to diminution of EDM action in machining mechanism. This finding is in agreement with micro-machining of copper plates, quartz glass and borosilicate glass using ECM and ECDM processes, respectively [29,30,31].

On the other hand, MRR indicates reverse trends with an increase in inter-electrode gap. The MRR significantly reduces with increase in inter-electrode gap (IEG) from level C1 to C3 (40 to 60 mm) as shown in Figure 3. Higher IEG leads to higher inter-electrode resistance, which in result reduces spark intensity in machining regime. This is because of increase in critical voltage required for hydrogen layer formation during machining. Therefore, at high inter-electrode gap maximum amount of energy is used for evaporation or heating of electrolyte rather than material removal mechanism. This tends to step down MRR as the inter-electrode gap increases. Similar behaviour was observed by machining of epoxy composite and ceramics using ECDM process [32,33]. From the Taguchi’s analysis, the optimal process parameters for MRR are A3B3C1, i.e., applied voltage 65 V, electrolyte concentration 30% and IEG of 40 mm respectively.

### 4.2. Overcut

**Figure 4:**
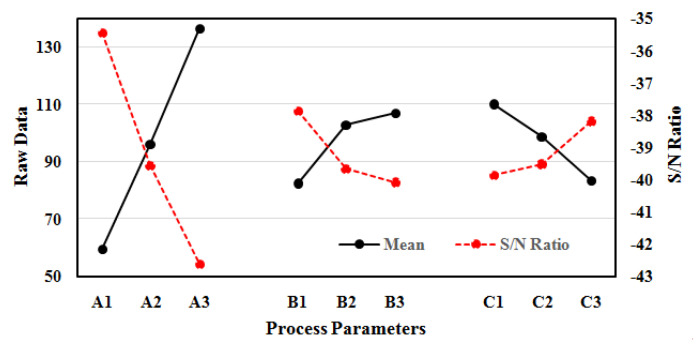
Raw data and S/N ratio plot for Overcut.

The reduction of machined hole overcut is highly desirable for miniaturized applications. Therefore, the analysis of process parameters on overcut is an important aspect in the domain of micro-machining. The influence of process parameters on overcut of machined holes is presented in Figure 4. The overcut of the machined hole increases sharply with an increase in applied voltage from level A1 to A3 (45 V to 65 V). The increase in applied voltage promotes side sparking on the periphery of tool electrode due to increase in size of hydrogen bubbles in machining zone. The large size hydrogen bubbles lead to generation of scattered spark over tool electrode surface, instead of consistent spark intensity. Therefore, the scattered spark phenomenon overcuts the machined hole with melting or uneven heating. This finding is in agreement with the machining of aluminium using a wire EDM process [34].

Similarly, the overcut of the machined hole sharply increases with increase in electrolyte concentration from level B1 to B2 (10% to 20%) and level B2 to B3 (20% to 30%). In comparison, the increasing rate of overcutting in stage B1 to B2 is slightly higher than stage B2 to B3. The high electrolyte concentration increases the electrical conductivity of solution, which tends to fasten the rate of hydrogen bubbles formation in ECDM process. The high density of hydrogen bubbles coalescence to form a thick film of hydrogen layer at the tool electrode surface. The puncture of thick hydrogen layer around tool electrode induced high discharge energy along the side walls of machining product. Therefore, the overcut of machined product increases due to uneven heating or melting. At higher concentration from level B2 to B3 (20% to 30%), the overcut of machined hole increases at low pace owing to the reduction of EDM action in machining mechanism. The similar behaviour is observed in the machining of SiC reinforced composite and glass using ECDM process [18,30,31].

On a positive note, the overcut of the machined hole reduces with high inter-electrode gap from level C1 to C3 (40 mm to 60 mm) as shown in Figure 4. The electrical conductivity of electrolyte decreases because of high inter-electrode gap raises the inter-electrode resistance. Moreover, the electrode potential required for hydrogen film formation increases with rising in the inter-electrode gap. This tends to slow down the side walls sparking in the machining zone. This occurs due to consumption of a greater amount of energy in Joule heating or evaporation of electrolyte for hydrogen film formation instead of machining mechanism. Therefore, the high inter-electrode gap is highly desirable for disposable of gases, electrolyte flow and minimization of overcut in the machined product. This finding is quiet similar to previous research work in the machining of epoxy composite and ceramics using ECDM process [32,33]. The optimum process parameters for overcut are A1B1C3, i.e., applied voltage 45 V, electrolyte concentration 10% and inter-electrode gap of 60 mm respectively.

### 4.3. Process Evaluation Using TOPSIS

TOPSIS technique is a well-recognised technique for multi-criteria decision making in different applications. The optimum solution by TOPSIS method is at smallest distance from ideal result and extreme distance from worst result [35]. The elementary steps involves in multi-response optimization by TOPSIS technique are as follows:

Step 1: The first step includes the illustration of output characteristics of ECDM in the organization of matrix. The developed matrix is known as decision matrix and represented in Table 3.

Step 2: This step includes creation of normalized matrix from decision matrix. Basically, the output in decision matrix has dissimilar units and magnitude. Therefore, the obtained normalized matrix (Nij) using equation 1 is presented in Table 3.

(1) $$N_{\mathrm{ij}}\;=X_{\mathrm{ij}}/[\sum_{j=1}^{M}{X_{\mathrm{ij}}}^{2}{]}^{2}$$

Step 3: Now, the weights are allocated to response variables depending on their relative importance. The weights are allocated in such a way that their summation is unity. For producing micro holes in CFRPC, the overcut has more importance than MRR. Therefore, the allocated weights for MRR and overcut are 0.4 and 0.6 respectively.

Step 4: This step includes multiplication of normalized matrix by respective weights to get weighted normalized matrix (Vij). The Vij is summarised using Equation (2) and represented in Table 3.

V_ij_ = w_ij_N_ij_ (2)

Step 5: This step defines the ideal and worst solution based on TOPSIS method. The ideal (V^+^) and worst (V^−^) can be calculated using Equations (3) and (4).

(3) $$V^{+}=\{(\sum_{i}^{\max}V_{\mathrm{ij}}\;/jƐJ).\;(\sum_{i}^{\min}V_{\mathrm{ij}}\;/jƐJ^{\prime }),\;i=1,2,3\ldots N\}=\{{V_{1}}^{+}.{V_{2}}^{+}.{V_{3}}^{+}\ldots \ldots {V_{M}}^{+}\}$$

(4) $$V^{-}=\{(\sum_{i}^{\min}V_{\mathrm{ij}}\;/jƐJ).\;(\sum_{i}^{\max}V_{\mathrm{ij}}\;/jƐJ^{\prime }),\;i=1,2,3\ldots N\}=\{{V_{1}}^{-}.{V_{2}}^{-}.{V_{3}}^{-}\ldots \ldots {V_{M}}^{-}\}$$

where J = (1, 2,……,M) represents the beneficial response and J′ = (1,2,……,M) known as non-beneficial response. The aim of this study is to increase MRR and minimize the overcut.

Step 6: Calculation of parting measures. The parting measures of every test trace from perfect and nastiest solution is calculated using Equations (5) and (6) and presented in Table 4.

(5) $${{Si}_{+}=[\sum_{j=1}^{n}(\mathrm{Vij}-{V_{j}}^{+}{)}^{2}]}^{1/2}$$

where i = 1,2,…m

(6) $${{S_{i}}^{-}=[\sum_{j=1}^{n}(\mathrm{Vij}-{V_{j}}^{-}{)}^{2}]}^{1/2}$$

where i = 1,2,…m

Step 7: Conclude the relative closeness index. The relative closeness to perfect solution is represented by Ci* and mathematically defined using Equation (7) and presented in Table 4.

C_i_* = S_i_^−^/(S_i_^−^ + S_i_^+^) (7)

where i = 1, 2,…m, (0 ≤ C_i_* ≤ 1)

Step 8: Arrange the relative closeness index either in increasing or decreasing order. The experiment trail with Ci* closest to one is hypothetically the best solution.

**Table 3:** Representation of decision matrix, normalized matrix and weighted normalized matrix for TOPSIS method.

Trial No.	Decision Matrix		Normalized Matrix N_ij_		Weighted Normalized Matrix V_ij_	
	MRR	Overcut	MRR	Overcut	MRR	Overcut
1	2.1150	49	0.3219	0.1573	0.1287	0.0944
2	2.1650	69	0.3295	0.2215	0.1318	0.1329
3	2.1765	61	0.3313	0.1958	0.1325	0.1175
4	2.1560	88	0.3281	0.2825	0.1312	0.1695
5	2.2210	80	0.3380	0.2569	0.1352	0.1541
6	2.2320	121	0.3397	0.3885	0.1359	0.2331
7	2.1456	110	0.3266	0.3532	0.1306	0.2119
8	2.2450	160	0.3417	0.5138	0.1366	0.3082
9	2.2478	140	0.3421	0.4495	0.1368	0.2697

**Table 4:** Relative closeness index and ranking by TOPSIS method.

Trial No.	Separation Measures		Relative Closeness Index C_i_*	Rank
	V^+^	V^−^		
1	0.0080	0.2138	0.9635	1
2	0.0388	0.1753	0.8185	3
3	0.0235	0.1907	0.8902	2
4	0.0753	0.1387	0.6480	5
5	0.0597	0.1542	0.7208	4
6	0.1387	0.0754	0.3523	7
7	0.1176	0.0963	0.4501	6
8	0.2138	0.0079	0.0356	9
9	0.1753	0.0393	0.1833	8

Table 4 displays the relative closeness index rankings of possible experiments. One is the rank given to the alternative first experimental test run, followed by 3 and 2. Table 5 displays the average values of the relative closeness index (Ci*) for a variety of input parameters. The TOPSIS technique designates the factor levels with the highest proximity index as the optimal parametric state. Therefore, the A1B1C3, i.e., Voltage 45 V, electrolyte concentration 10% and inter-electrode gap of 60 mm were observed as optimum process parameters. The analysis of variance (ANOVA) for Ci* value is performed to evaluate significant process parameters. The ANOVA of Ci*values is represented in Table 6. It reveals that Ci* value is dominantly affected by voltage followed by IEG and electrolyte concentration of ECDM process.

### 4.4. Confirmatory Test and Morphology of Drilled Hole

The TOPSIS approach of multi-response optimization was used to perform a confirmatory test analysing the ensuing improvement in quality parameters. TOPSIS’s best parameter settings (voltage 45 V, electrolyte concentration 10%, and inter-electrode gap 60 mm) were obtained with the following values: A1B1C3. These experimental conditions were used for conformation test. The alternative experiment no. 8 with lowest relative closeness index (Ci*) used as preliminary parametric setting. The results of confirmatory test and initial parametric condition are shown in Table 7. The confirmatory test concluded that overcut of machined hole decreases from 150 µm to 48 µm. Additionally, the MRR marginally decrease with optimum parametric conditions of TOPSIS method. As discussed in the above section, the overcut is domineering output characteristic as equated to MRR in machining of miniaturized products. Therefore, optimum parametric condition obtained by TOPSIS method is validated for producing micro holes.

**Figure 5:**
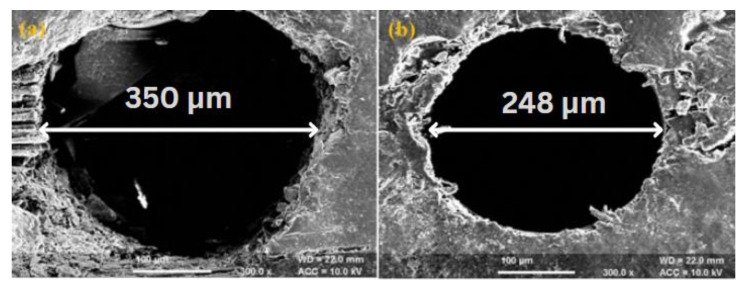
SEM of drilled micro-holes using (**a**) Taguchi’s methodology (**b**) TOPSIS Method.

**Figure 6:**
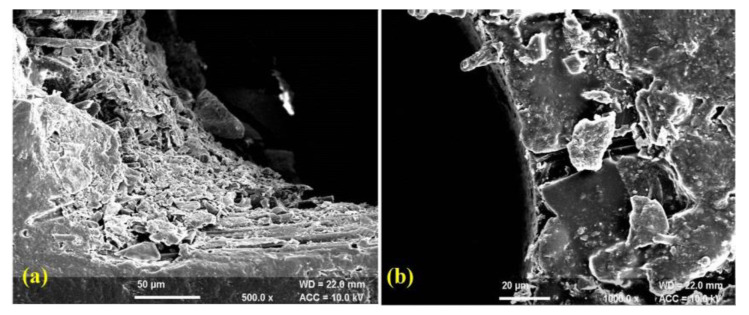
SEM of drilled micro-hole wall surface (**a**) Taguchi’s methodology (**b**) TOPSIS Method.

The morphological behaviour of machined sample reveals that work piece machined with optimum parameter conditions of TOPSIS method produces better quality machined surface and uniformity in diameter as compared to Taguchi’s methodology. The scanning electron microscopy (SEM) of drilled micro-holes is shown in Figure 5a,b. The SEM micrograph (Figure 5a) indicates the presence of heat affected zones (HAZ), uneven cutting and heating of fibres with small micro-cracks. On the other hand, the HAZ, uneven cutting of fibres and micro-cracks can be reduced up to a significant level with TOPSIS method (Figure 5b). Likewise, the SEM micrographs of drilled hole wall surface is presented in Figure 6a,b. The drilled hole wall surface is non-uniform with smalldebris,uneven cutting of fibres as shown in Figure 6a. Additionally, the wall surface of hole machined with TOPSIS method signifies uniform boundary surface with minor irregularities (Figure 6b). Therefore, it is concluded that tearing, uneven cutting of fibres, micro-cracks and irregularities over machined surface can be effectively controlled with TOPSIS method.

## 5. Conclusions

This study is evident of ECDM potential for producing micro holes on CFRP Composites. The numerous conclusions have been drawn from this study are following as:

1. Using the TOPSIS approach, the optimal parameters for creating micro holes in CFRP composite were determined to be: voltage 45 V, electrolyte concentration 10%, and inter-electrode spacing 60 mm.
2. When compared to electrolyte concentration, the voltage and inter-electrode gap were found to be the most important process parameters that influence the output quality characteristics.
3. Increases in applied voltage and electrolyte concentration, while decreases in inter-electrode gap, both have a direct and positive effect on MRR. The ratio of overcut to input values is also roughly the same.
4. According to Taguchi’s analysis, the best input process parameters for MRR and overcut are A3B3C1 and A1B1C3, which stand for an applied voltage of 65 V and 45 V, an electrolyte concentration of 30% and an inter-electrode gap of 40 mm and 60 mm, respectively.
5. Uneven fibre cutting, microcracks, and minute debris were all visible on the micrograph taken by the SEM across the boundary walls of the machined surface.
6. SEM micrograph of machined sample shows improved surface quality and reduced imperfections because of TOPSIS.
7. The comparative analysis shows integrated Taguchi-TOPSIS methodology can be effectively used for generating micro holes in fibrous and electrically semi-conductive materials.

## Figures and Tables

**Table 1:** Process parameters and their Levels.

Symbols	Process Parameters	Level 1	Level 2	Level 3
A	Voltage (Volts)	45	55	65
B	Electrolyte Concentration (wt.%/V)	10	20	30
C	Inter-Electrode Gap (mm)	40	50	60

**Table 2:** Obtained Results for Taguchi’s Methodology.

Trial. No.	Voltage	Electrolyte Concentration	Inter Electrode Gap	MRR (mg/min)		Overcut (µm)	
				Mean	S/N Ratio	Mean	S/N Ratio
1	45	10	40	2.1150	6.50621	49	−33.8039
2	45	20	50	2.1650	6.70916	69	−36.7770
3	45	30	60	2.1765	6.75517	61	−35.7066
4	55	10	50	2.1560	6.67298	88	−38.8897
5	55	20	60	2.2210	6.93097	80	−38.0618
6	55	30	40	2.2320	6.97388	121	−41.6557
7	65	10	60	2.1456	6.63098	110	−40.8279
8	65	20	40	2.2450	7.02433	160	−44.0824
9	65	30	50	2.2478	7.03515	140	−42.9226

**Table 5:** Mean of Ci*values at different levels.

Levels	Process Parameters		
	A	B	C
1	0.8907	0.6872	0.4505
2	0.5737	0.5250	0.5499
3	0.2230	0.4753	0.6870
Delta	0.6677	0.2119	0.2366
Rank	1	3	2

**Table 6:** ANOVA for Ci* values by TOPSIS method.

Source	DF	Adj SS	Adj MS	F Value	*p* Value
A	2	0.66937	0.334684	40.32	0.024
B	2	0.07371	0.036853	4.44	0.184
C	2	0.08465	0.042327	5.10	0.164
Error	2	0.01660	0.008300		
Total	8	0.84433			

**Table 7:** Confirmatory test by TOPSIS method.

Responses	Initial Parametric Condition (A_3_B_2_C_1_)	Optimum Parametric Condition (A_1_B_1_C_3_)
MRR (mg/min)	2.232	2.1267
Overcut (µm)	150	48

## Data Availability

The authors confirm that the data supporting the findings of this study are available within the article.
